# Decades of Tight Fiscal Policy Have Left the Health Care System in Italy Ill-Prepared to Fight the COVID-19 Outbreak

**DOI:** 10.1007/s10272-020-0886-0

**Published:** 2020-06-07

**Authors:** Franz J. Prante, Alessandro Bramucci, Achim Truger

**Affiliations:** 1 Institute for International Political Economy Berlin, Berlin School of Economics and Law, Badensche Straße 50-51, 10825 Berlin, Germany; 2 Institute for Socio-Economics, University Duisburg-Essen, Forsthausweg 2, 47057 Duisburg, Germany

## Abstract

Although austerity was particularly strong in the aftermath of the economic crisis of 2008 and its consequences in the euro area, Italian fiscal policies have been characterised by tough consolidation periods ever since the 1990s.

The Sars-CoV-2 pandemic and the resulting COVID-19 disease is overwhelming some European health systems in an unprecedented manner. The situation remains particularly serious in Italy. In the most affected regions, the Italian national health system (*Sistema sanitario nazionale*, SSN) has been unable to cope with the care of COVID-19 patients. This article takes a closer look at the connection between health care and strong budget consolidation in the case of Italy. Although austerity was particularly strong in the aftermath of the economic crisis of 2008 and its consequences in the euro area, Italian fiscal policies have been characterised by tough consolidation peri-

**Franz Prante**, Berlin School of Economics and Law (HWR); and University of Duisburg-Essen, Germany.

**Achim Truger**, University of Duisburg-Essen; and German Council of Economic Experts, Wiesbaden, Germany.

* This contribution is based on preliminary parts of an ongoing research project on the Italian economy funded by Friedrich-Ebert-Stiftung in Germany.

Figure 1

**Government and compulsory health care expenditure per capita and primary balance in Italy**

constant prices (2010) and percent of potential GDP

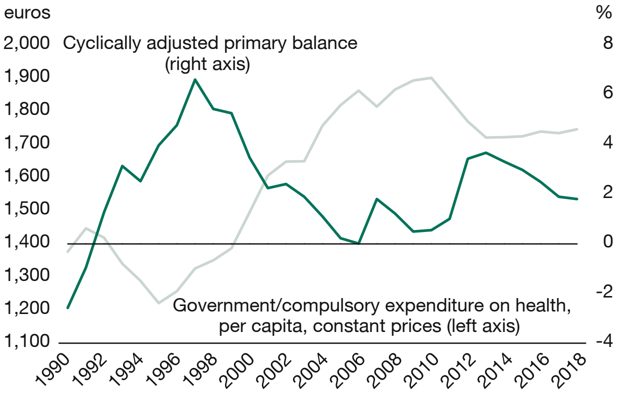

Notes: the OECD classifies government funded and compulsory insurance funded health care expenditures into the same category.

Sources: OECD; IMF.

Figure 2

**Government and compulsory health care expenditure per capita in selected countries**

constant prices (2010)

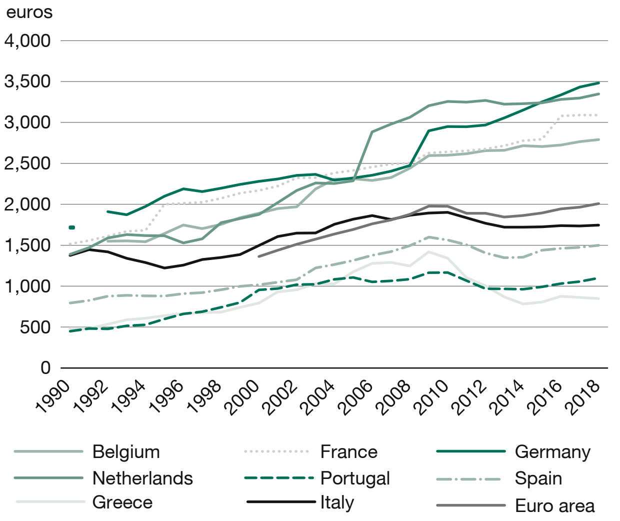

Notes: Data for Malta and Cyprus are not available and are not included in the euro area average. Euro membership as of 2020. Breaks present in the data. For 2018, provisional data or OECD estimates.

Sources: OECD; authors' calculations.

ods ever since the 1990s. Over the years, the SSN has undergone a profound transformation aimed at containing costs and increasing efficiency. The question now is whether the consequences of these measures have left the SSN unprepared to face the scourge of COVID-19.

Italy's SSN was founded in 1978. Based on the national constitution (Article 32), the state guarantees the universal right and largely free access to health care services.[1] During the 1990s, a first series of far-reaching reforms was implemented in an attempt to contain costs in the face of the growing healthcare needs of an ageing population and rapidly improving technologies (Pavolini and Vicarelli, 2013). These reforms were largely in line with the market-liberal 'New Public Management' approach and their primary objective was to limit Italy's public deficits and debt (Pavolini and Vicarelli, 2013). Cost containment was therefore motivated by the macroeconomic context of the time, characterised by Italy's efforts to meet the Maastricht criteria and the requirements of the Stability and Growth Pact, which led to an overall tightening of public spending. More recently, the global financial crisis and the policy response to the euro crisis put a further strain on the Italian economy and significant restrictions on health care spending returned to the national agenda (De Belvis et al., 2012). Since the early 1990s, the Italian government has registered almost 30 consecutive years of primary budget surpluses (Figure 1). This signals that what the government extracted from the national economy in terms of taxes has been larger than what people received in public services for almost three decades. Figure 1 also shows that periods with cuts in real health care expenditure tend to correspond with or follow periods of strong budget consolidation in the first half of the 1990s and in the euro crisis after 2010.

The development of Italian health care expenditure is reported in Figure 2 together with data for selected European countries and the euro area average. Three phases can be observed in the evolution of Italian expenditure. In the 1990s, unlike most other industrialised countries, Italy experienced a decline in public and compulsory health care expenditure (measured in constant euros per capita). It was not until the end of the 1990s that a slight upward trend began when spending increased in parallel to the other European countries until the late 2000s. From 2010 onwards, a new phase of spending containment began, lasting until 2015. In this period, public health care spending was similarly affected in Portugal and Spain and to a larger extent in Greece, i.e. the countries hardest hit by the euro crisis and the subsequent austerity policies. By contrast, in this period a rapid increase in public and compulsory healthcare spending per capita took place in Germany, France and Belgium.

1 The European Commission (2019) in its Country Report Italy 2019 finds the SSN to be generally efficient and its outcome in terms of health indicators good, albeit with regional disparities in the provision of health services affecting equity and efficiency.

Figure 3

**Percentage change in government and compulsory health care expenditure per capita in selected countries**

constant euros (2010)

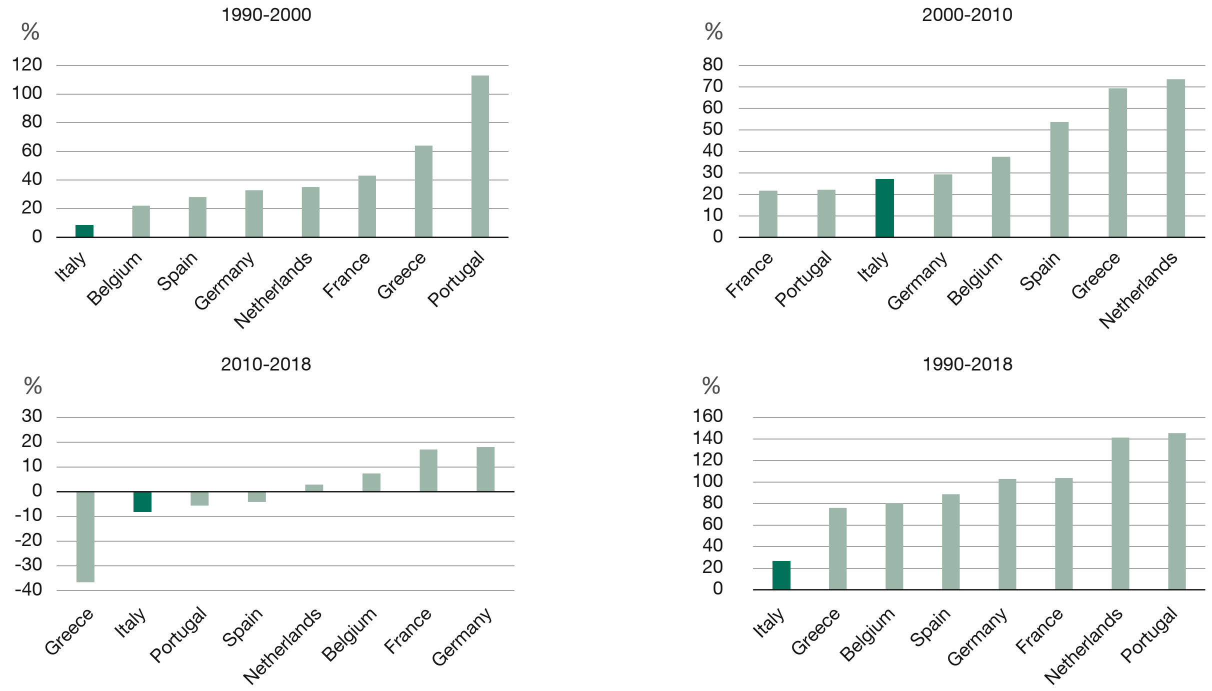

Notes: For Belgium, the value for 1990 is missing and was replaced with the value for 1993.

Sources: OECD; authors' calculations.

Figure 3 shows the percentage change in government and compulsory health care per capita for these three different phases and for the entire period between 1990 and 2018. From 1990 to 2000, a first phase of expenditure containment took place in Italy, in which public expenditure increased by only 8.7%. After a slightly expansive second phase from 2000 to 2010, in which spending per capita in Italy increased by 27.1%, the growth of public health expenditure registered a reduction in the third time interval (as was the case in Portugal, Greece and Spain). In this period, characterised by the most recent set of budget cuts, per capita spending in Italy decreased by 8.2% – less strongly than in Greece but more than in Spain and Portugal. In contrast, the group of Northern countries registered an increase. Altogether, from 1990 to 2018, public and compulsory health care expenditure per capita in Italy increased by less than 26.8%, which is by far the lowest value among the European countries reported in Figure 3.

In the last decade, the extent of cuts in the SSN was particularly dramatic. In the wake of the financial and economic crisis of 2008, total public healthcare expenditure in Italy (including investments, intermediate consumption, research and development and other components) has suffered a drastic setback. From 2008 to 2018, total public health care expenditure in nominal terms (i.e. including inflation) increased by only 5.3% in Italy, while in Germany it increased by 46.8% (Figure 4a). Moreover, COFOG[2] data provides evidence of the extent of cuts in hospital services.[3] Unlike Northern European countries, Italy (together with Portugal and even more so Greece) has reduced public spending for hospital services.

From 2011 to 2018, cuts in public hospital services have contributed substantially to the negative dynamics of the percentage growth rate of the total public expenditure for health care (Figure 4b). Although austerity policy placed a great burden on the health care system, the share of health care expenditures in total government spending

2 The Classification of the Functions of Government (COFOG) health aggregate (GF07 and relative groups) classifies all types of government expenditure for the purpose of health (including expenditure on employees, intermediate consumption, government expenditure on gross capital formation, etc.). The delineation of government expenditure in the COFOG classification differs from the System of Health Accounts.

3 According to the COFOG classification, day hospitalisation is classified under hospital services.

Figure 4a

**Percentage change of total government spending for health care and for hospital services, 2008-2018**

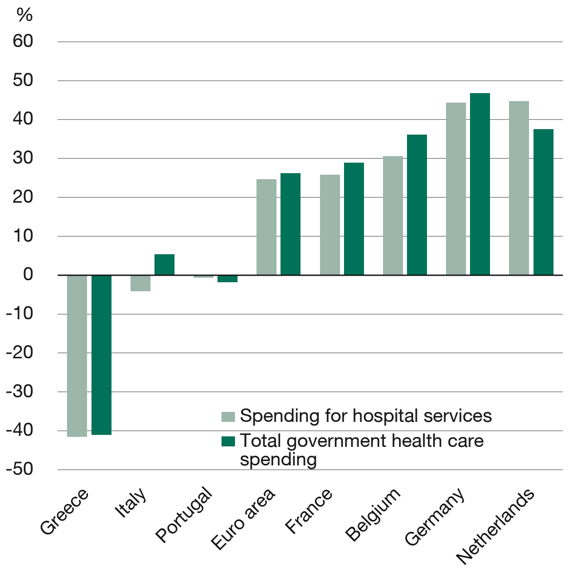

Sources: Eurostat COFOG; authors' calculations.

Figure 4b

**Composition of the growth rate of total government spending for health care in Italy, nominal values**

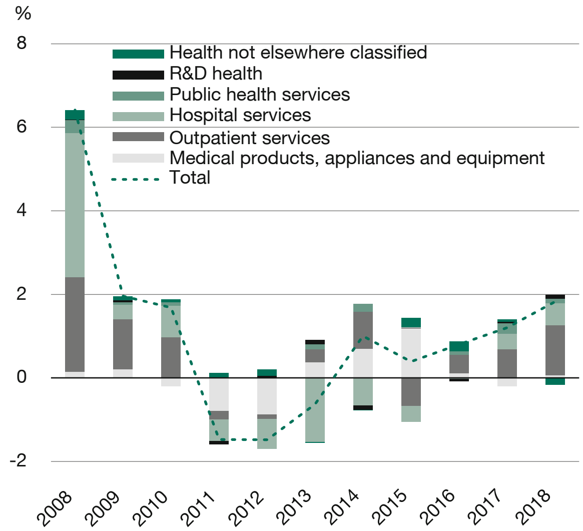

Note: Second level COFOG.

Sources: Eurostat COFOG; authors' calculations.

has increased from 10% in 1995 to 14.7% in 2008 and remained above 14% after 2008, according to COFOG data. This may indicate that health care provision was important to the government despite general spending constraints. However, this did not prevent Italian expenditures from falling below the international trend.

In the EU, almost one-third of public health care expenditure is used to cover the running expenses of inpatient curative institutions (European Hospital and Healthcare Federation, 2018). Over the years, hospitals have been subject to increasing pressure and have often been seen as a major potential source for cuts in public health systems (see McKee, 2004; Popic, 2020). Cost containment strategies revised the use and provision of inpatient hospital care in favour of day hospital and outpatient services, thereby consistently sacrificing hospital capacity. Data from the World Health Organization (WHO) show that since the beginning of the 1990s, the number of hospitals has been drastically reduced throughout Europe, but particularly in Belgium and Italy. Acute care hospitals are currently a central element in the fight against COVID-19. A higher number of acute care hospitals could have also facilitated the isolation of infected patients, reducing the risk of contagion. Figure 5 shows that after starting from a level similar to Germany in 1990, Italy has reduced per capita hospital capacity much more than many other countries within two decades. From 2010 on, the number of acute care hospitals in Italy fell below the EU average. The trend continued to decline throughout the years of the euro crisis.

The availability of acute care beds was reduced even more drastically than hospital capacity (Figure 6). Although a pronounced trend towards reducing acute care beds can be observed in many European countries, few European countries have reduced the number of available beds as much and to such a low level as Italy. In 1990, Italy had seven beds per 1,000 inhabitants, a value close to Germany and above the EU average. In 2017, the number of acute care beds had dropped to 2.6 per 1,000 inhabitants, significantly lower than in Germany with six beds available per 1,000 persons and much closer to the historically low value of Spain. Thus within a rather short time period, Italy found itself at the lower end of the spectrum in Europe.

There is also a considerable difference in the provision of intensive care beds, with Italy again at the tail end in Europe (Rhodes et al., 2012; OECD, 2020). Although in recent years the number of intensive care beds in Italy has remained relatively constant (Figure 7), intensive care capacity has not been expanded (in contrast to e.g. Germany) despite warnings of possible bottlenecks in accommodation capacity of intensive care patients (Rhodes et al., 2012).

Figure 5

**Acute care hospitals per 100,000 inhabitants, eight largest EMU countries and EU average**

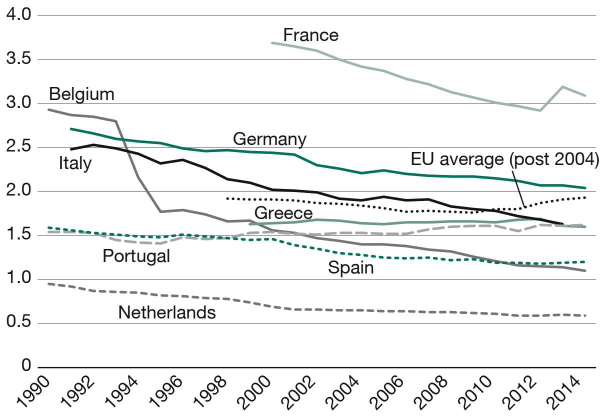

Sources: WHO; authors' calculations.

Figure 6

**Acute care beds per 1,000 inhabitants, eight largest EMU countries and EU average**

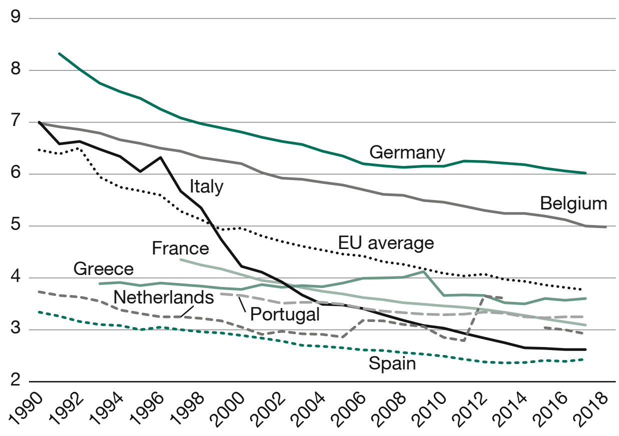

Note: EU membership as of 2020 with country data available.

Sources: OECD; authors' calculations.

Italy, as well as other European countries, would have been better prepared for adequate treatment of COVID-19 patients in severe and critical condition if the capacity of acute and emergency care had not been reduced. Oxygen-assisted beds are particularly relevant for the inpatient treatment of COVID-19. For some patients, breathing difficulties worsen in the course of the illness, making intensive medical care necessary. The public discussion therefore focuses primarily on the availability of intensive care capacities and mechanical ventilation equipment. However, the speed at which machine ventilation should be provided is a matter of controversy among lung specialists and intensive care physicians (Gattinoni et al., 2010). The current research gap on COVID-19 may therefore also require a comprehensive diagnosis of patients by lung specialists, which could lead to better treatment outcomes (see also Begley, 2020). In this context, the substantial reduction in the number of pneumological beds during the phase of intensified austerity after 2010 in Italy is particularly tragic. According to the data of the Italian Ministry of Health, the number of pneumological beds has decreased from 4,414 in 2010 to 3,573 in 2018 – a reduction of 19%.

Figure 7

**Total number of intensive care beds and pneumological beds in Italy and Germany**

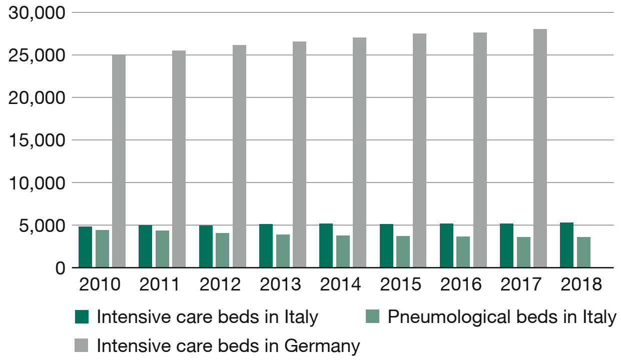

Sources: Italian Ministry of Health; Destatis.

The reduction of resources in the public health system and in particular in the operation of public hospitals in Italy has been going on for almost 30 years. The Italian population is currently paying the price of prolonged tight budget policies in the SSN. The one-sided focus on fiscal constraints and debt reduction has deprived the Italian health sector of an important part of its capacity to offer adequate protection to the population. The sizeable reduction of resources has caused severe difficulty to the SSN in effectively tackling the consequences of COVID-19. The outbreak of the health crisis has sounded a wake-up call that cannot remain unheard.
